# Neurochemical differences in core regions of the autistic brain: a multivoxel ^1^H-MRS study in children

**DOI:** 10.1038/s41598-024-52279-x

**Published:** 2024-01-29

**Authors:** Ana Dionísio, Ana Espírito, Andreia C. Pereira, Susana Mouga, Otília C. d’Almeida, Guiomar Oliveira, Miguel Castelo-Branco

**Affiliations:** 1 Coimbra Institute for Biomedical Imaging and Translational Research (CIBIT), University of Coimbra, Pólo das Ciências da Saúde, Azinhaga de Santa Comba, 3000-548 Coimbra, Portugal; 2 Institute of Nuclear Sciences Applied to Health (ICNAS), University of Coimbra, Pólo das Ciências da Saúde, Azinhaga de Santa Comba, 3000-548 Coimbra, Portugal; 3 Faculty of Medicine, University of Coimbra, Pólo das Ciências da Saúde, Azinhaga de Santa Comba, 3000-548 Coimbra, Portugal; 4 Centro de Desenvolvimento da Criança, Unidade de Neurodesenvolvimento e Autismo, Hospital Pediátrico, Centro Hospitalar e Universitário de Coimbra, Coimbra, Portugal; 5 Faculty of Medicine, University Clinic of Pediatrics, University of Coimbra, Coimbra, Portugal

**Keywords:** Neuroscience, Autism spectrum disorders

## Abstract

Autism spectrum disorder (ASD) is a neurodevelopmental condition which compromises various cognitive and behavioural domains. The understanding of the pathophysiology and molecular neurobiology of ASD is still an open critical research question. Here, we aimed to address ASD neurochemistry in the same time point at key regions that have been associated with its pathophysiology: the insula, hippocampus, putamen and thalamus. We conducted a multivoxel proton magnetic resonance spectroscopy (^1^H-MRS) study to non-invasively estimate the concentrations of total choline (GPC + PCh, tCho), total N-acetyl-aspartate (NAA + NAAG, tNAA) and Glx (Glu + Gln), presenting the results as ratios to total creatine while investigating replication for ratios to total choline as a secondary analysis. Twenty-two male children aged between 10 and 18 years diagnosed with ASD (none with intellectual disability, in spite of the expected lower IQ) and 22 age- and gender-matched typically developing (TD) controls were included. Aspartate ratios were significantly lower in the insula (tNAA/tCr: p = 0.010; tNAA/tCho: p = 0.012) and putamen (tNAA/tCr: p = 0.015) of ASD individuals in comparison with TD controls. The Glx ratios were significantly higher in the hippocampus of the ASD group (Glx/tCr: p = 0.027; Glx/tCho: p = 0.011). Differences in tNAA and Glx indices suggest that these metabolites might be neurochemical markers of region-specific atypical metabolism in ASD children, with a potential contribution for future advances in clinical monitoring and treatment.

## Introduction

Autism spectrum disorder (ASD) is a neurodevelopmental condition with a heterogeneous clinical presentation, affecting different cognitive and behavioural domains. ASD is primarily characterized by difficulties with social interaction and communication, the presence of restrictive and stereotyped behaviours as well as sensory processing difficulties^1^. Although research has made it possible to increase knowledge about possible etiological factors, understanding the neurobiological basis of ASD is still a major challenge^2^.

Proton magnetic resonance spectroscopy (^1^H-MRS) is a non-invasive magnetic resonance (MR)-based technique that provides information about the in vivo biochemistry of the human brain by estimating the concentrations of brain metabolites^3^. The most commonly studied metabolites are N-acetyl-aspartate (NAA), choline-containing compounds (tCho), glutamate + glutamine (Glx), and creatine + phosphocreatine (tCr)^4^. These molecules play specific roles in brain metabolism and are important indicators of neuronal integrity and/or proliferation, neurotransmission and/or metabolism, and cellular energy homeostasis^4–6^.

Several studies have suggested that brain metabolite concentrations are different in ASD as compared with neurotypical participants, although findings remain inconsistent^3,7–11^.

The controversy present in the ^1^H-MRS literature in ASD motivated us to contribute to this issue by developing a study investigating four regions relevant to the pathophysiology of ASD: the insula, hippocampus, thalamus and putamen. We selected these regions of interest due to the differences found in ASD in terms of functional anatomy and connectivity and their relation to core symptoms^10,12^. Despite this fact, they have been scarcely studied and we did not find any study considering the four regions simultaneously in the same individuals.

There is for all these regions some evidence of pathophysiological abnormalities in ASD. The insula is a core region of the saliency network involved in visceral and somatosensory processes, autonomic control, emotional experience, social cognition, decision making, attention and salience processes^13–15^. In agreement with this broad array of functions, it has strong functional connectivity to various other cortical and subcortical areas^13,14,16^. Structural studies in ASD individuals have found alterations in the insula volume^17^, sulcus depth^18^, and gyrification^19^. Functional abnormalities include reduced regional cerebral blood flow^20^, decreased (including intrinsic) functional connectivity^21–24^, atypical activation^25^, diminished regional homogeneity^26^, and localized changes in subregional organization^27^. Finally, activation patterns of the insula can potentially distinguish individuals with ASD from typically developing (TD) children^28^.

The hippocampus plays a critical role in learning and memory^29–31^ and is worth studying in ASD because of the known impairments in episodic memory^32^. The investigation of the hippocampal volume has revealed inconsistent results, with the literature pointing out decreased^33^, increased^34^, or unchanged^35^ volumes in ASD. Studies have also found functional abnormalities such as decreased functional connectivity in episodic memory retrieval tasks^36^. In addition, *postmortem* research reported increased cell density and abnormally small cells in the hippocampus in ASD^37^.

The thalamus is a core relay structure^38–40^. Atypical thalamocortical connectivity is the predominant finding in ASD studies^41^. Many authors have described hyperconnectivity of the thalamus in children and adults^41–44^. In addition, differences in the surface area and shape of the thalamus of ASD individuals have also been reported^45^. Alterations in this region may help explain some autism-related symptoms due to differences in sensorimotor processing in ASD^43,44^.

The putamen is involved in cognitive (anterior putamen) and sensorimotor processes (posterior putamen)^46^. Several studies have found increased volume^47^, asymmetry^48^, and altered connectivity in the putamen^49^ in ASD. These regional abnormalities have been associated with core symptomatology. Accordingly, this region likely plays a role in repetitive and restricted behaviours as well as impaired social interactions in ASD^47,48^.

### ^1^H-MRS findings in the insula, hippocampus, thalamus, and putamen

In particular, with respect to the ^1^H-MRS findings in these four regions the following findings were reported: (1) Lower levels of tCr^50^ in the left insula and tNAA^51^ in the insula. (2) Lower tNAA or tNAA /tCr^52–54^, higher tCr^55,56^ and Glx^55^, as well as higher tCho^56^ and tCho/tCr^54^ were described in the hippocampal-amygdala complex; however, other studies reported higher tNAA and tNAA /tCr^57^ in the same region. (3) Lower tNAA (or tNAA ratios)^50,58,59^, tCr^50,58^, tCho^50,58^, and Glx^59,60^ have been found in the thalamus. (4) Higher Glx/Cr^61^ and lower tNAA^50^ were found in the putamen.

In spite of the available ^1^H-MRS literature, these regions remain understudied, in particular the insula, and findings seem inconsistent. Here we present a ^1^H-MRS study in children diagnosed with ASD. The main objective was to quantify metabolite levels and compare them between groups (ASD and controls), taking advantage from a multivoxel approach that allows the scanning of multiple regions simultaneously as well as the selection of small volumes targeting specific anatomical regions. Based on previous reports, we expected to find altered metabolites in ASD patients in these regions.

## Results

Groups were matched for age (t(42) = 0.324, p = 0.748) and handedness (p = 0.233). ASD participants had all FSIQ > 70, defined as absence of intellectual disability by ICD10. However, they presented the expectedly lower intellectual quotient compared with typically developing controls (full-scale IQ: t(42) = − 5.841, p < 0.001; verbal IQ: t(42) = − 6.449, p < 0.001; performance IQ: U = 148.5, p = 0.027).

In the following sections, we present the between-group comparisons for the metabolites in the selected regions-of-interest.

### Insula

**Figure 1:**
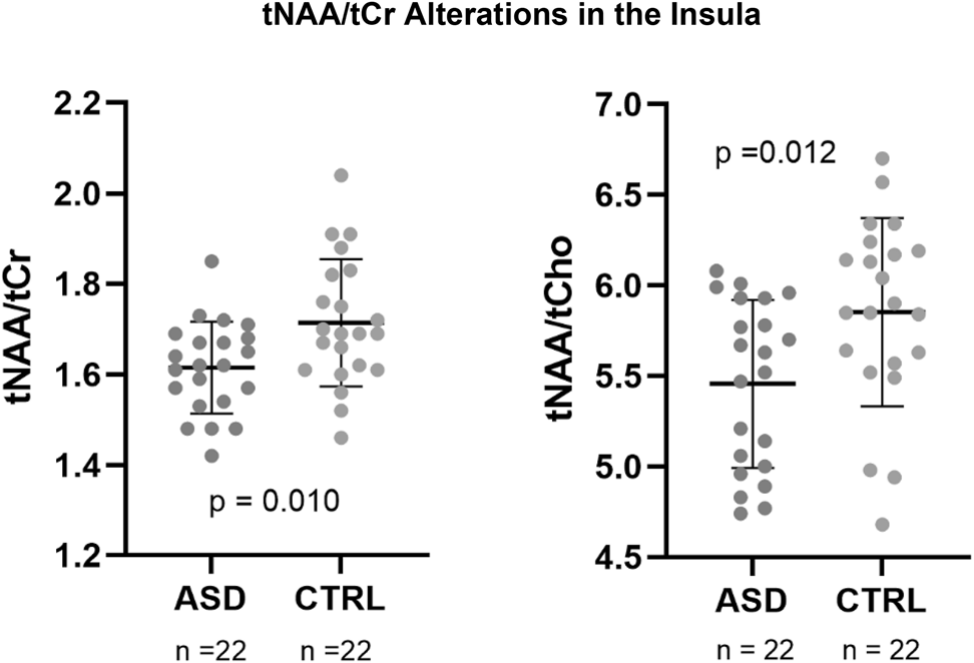
Differences in tNAA/tCr and tNAA/tCho between autism spectrum disorder (ASD) and control (CTRL) groups (mean ± SD).

Total N-acetyl-aspartate ratios in the insula were significantly lower in the ASD group, both as assessed by tNAA/tCr (t(42) = − 2.696, p = 0.010) and by tNAA/tCho (U = 136.0, p = 0.012), as observed in Fig. 1. Total choline and Glx ratios, in turn, did not differ between groups (p ≥ 0.05). Please note that the analysis of metabolite-to-tCho ratios, which has limitations due to the larger variability of Cho in psychiatric diseases, is secondary as it serves mainly as a replication to test the hypothesis whether some of the effects are generalizable even when the method of ratio calculation is changed.

### Hippocampus

**Figure 2:**
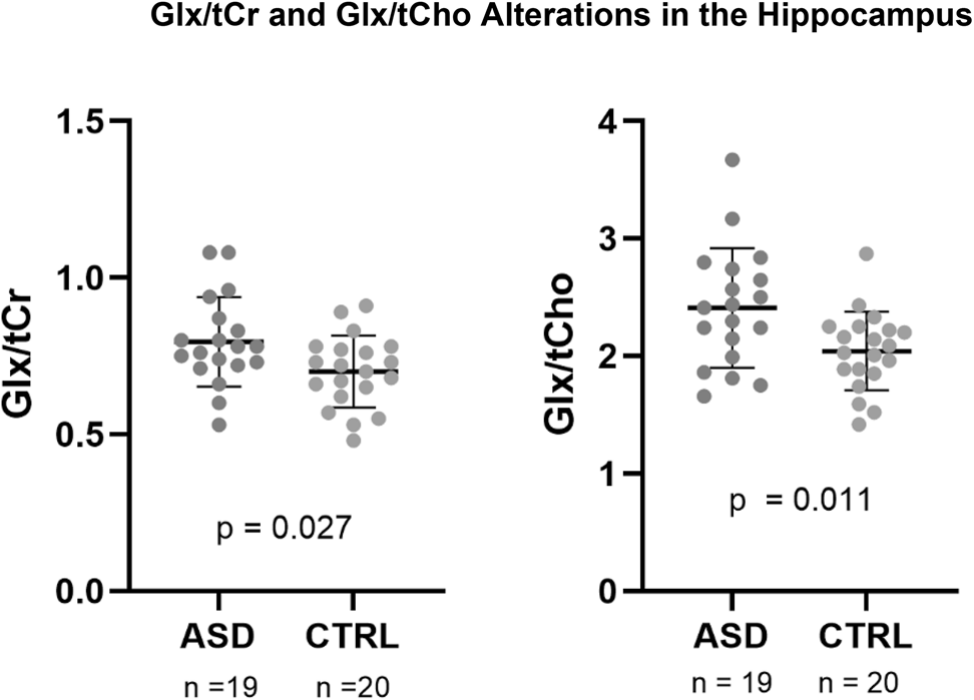
Differences in Glx/tCr and Glx/tCho between autism spectrum disorder (ASD) and control (CTRL) groups (mean ± SD).

The Glx/tCr (t(37) = 2.302, p = 0.027, Fig. 2) and Glx/tCho (t(37) = 2.691, p = 0.011, Fig. 2) in the hippocampus were significantly higher in the ASD group. Total choline and tNAA ratios, in turn, did not differ between groups (p ≥ 0.05).

### Putamen

**Figure 3:**
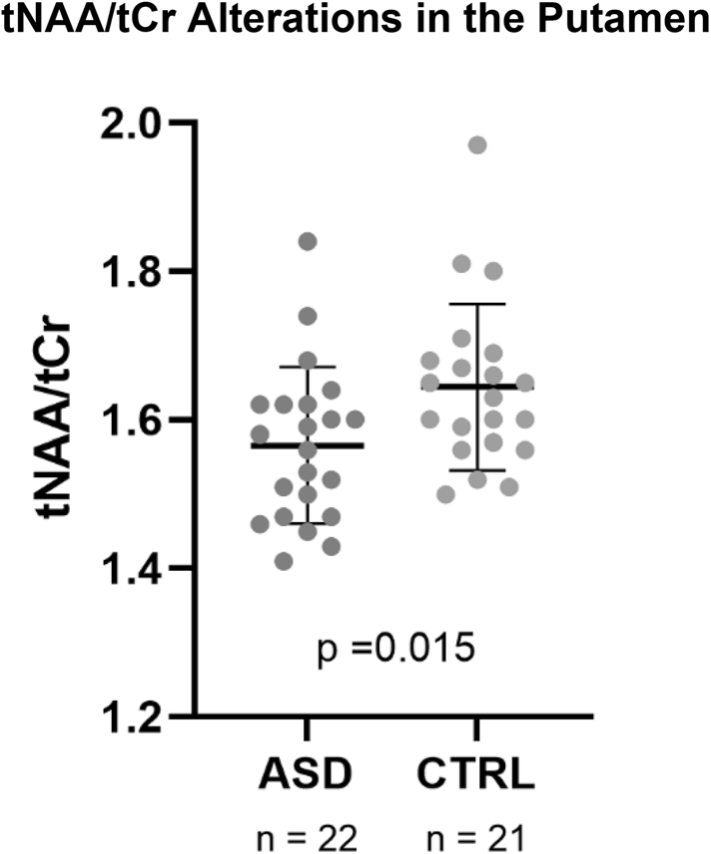
Differences in tNAA/tCr between autism spectrum disorder (ASD) and control (CTRL) groups (mean ± SD).

The tNAA/tCr ratio in the putamen was significantly lower in the ASD group (U = 131.5, p = 0.015, Fig. 3). Using tCho as reference only a trend level between-group difference was present (t(41) = − 1.829, p = 0.075). Total choline and Glx ratios, in turn, did not differ between groups (p ≥ 0.05).

### Thalamus

Regarding the thalamus, we did not detect significant between-group differences for any of the metabolites under investigation in this work.

In general, excluding medicated participants did not change the patterns of results except for the putamen where results became only marginally significant (Supplementary Table S1).

### Correlations between altered metabolite ratios and intellectual quotient and autism diagnostic scores

In the insula, the tNAA/tCr was negatively correlated with verbal IQ (r = − 0.474, p = 0.026) in the ASD group and with performance IQ in TD controls (rs =− 0.465, p = 0.029). In the hippocampus, we did find a negative correlation between the total scores of ADOS and Glx ratios (Glx/tCr: r = − 0.564, p = 0.012; replicated for Glx/tCho: r = − 0.595, p = 0.007) in the ASD group. We also found a correlation of tNAA/tCho measures between the putamen and insula: rs = 0.717, p < 0.001.

### Voxel tissue composition and quality of the ^1^H-MRS data

No statistically significant differences were observed in the tissue composition, including WM, GM or CSF, comparing ASD with controls in any region but hippocampus (% CSF: U = 158.0, p = 0.049) and thalamus (% WM: t(42) = 2.103, p = 0.042), which showed only marginal differences. We present the tissue proportions in the voxels selected for analyses in each region both for patients and controls in Supplementary Table S2.

We assured that all included metabolites had a Cramér-Rao Lower Bound (CRLB) ≤ 20%, which is normally used as reliability measure of metabolite concentration. We report the values of other spectral quality parameters (full-width at half maximum, FWHM and signal-to-noise ratio, SNR) in Supplementary Table S3, for completeness and following recent reporting guidelines^62^.

## Discussion

This work allowed to provide further insight into the neurochemical differences involved in ASD in different brain regions. This is important for tailoring new therapeutic interventions that may change different regions in a distinct manner. Here, we studied whether tNAA and Glx ratios to tCr (and whether they could be replicated concerning ratios to tCho), differ in a selection of brain regions that have been described with anatomical and functional abnormalities in ASD, specifically the insula, hippocampus, thalamus and putamen.

The levels of tNAA/tCr were significantly lower in the ASD group both in the insula and in the putamen. Moreover, the ratio to choline (tNAA/tCho) was also significantly lower in the insula. In the hippocampus we observed significantly higher Glx, either using the ratio to total creatine (Glx/tCr) or choline-containing compounds (Glx/tCho). Considering the thalamus, we did not observe any neurochemical between-group differences.

To our knowledge, only one previous study found lower tNAA in the insula^51^. Friedman et al. in turn, found no differences in tNAA in the insula, but, in line with our results, they also reported lower absolute metabolite levels in the left putamen^50^. On the other hand, the tNAA differences previously observed in the thalamus^50,58^ were neither replicated in this study nor by others^63^. N-acetyl aspartate is a brain metabolite synthesized in the neurons (particularly in mitochondria) and catalyzed in the glia^4^, being considered an indicative of neuronal density and integrity^3^ and/or mitochondrial function^64^ explaining its relation to multiple pathological conditions^65^. Decreased neuronal or axonal density and impaired mitochondrial metabolism are putative explanations for the lower tNAA in ASD^3,66^. Differences in tNAA signals in both the insula and putamen, which are connected from the functional and anatomical points of view, suggests future additional studies relating neurochemistry with known alterations in functional connectivity^21–24,49^.

Our observation that tNAA/tCr is not different in the hippocampus, is consistent with previous studies^55,56,67,68^. Nevertheless, other authors found higher^57^ or lower^52,54^ tNAA in this region in ASD. While some of them analyzed the absolute concentration^56,67,69^, others used the ratio to tCr as a measure^54^. In these studies, altered tNAA indices were found only in children^52,54,57^ and in some cases only for one hemisphere^52,57^. In any case, and in spite of some discrepancies, tNAA seems to be a consistently altered metabolite in ASD.

As far as we are aware, we report for the first time Glx/tCr alterations in the hippocampus alone in children and adolescents with ASD. These measures were higher in the ASD group which is in line with the findings in the right hippocampus for the adult ASD population reported as absolute quantification^55^. Glutamate, the main excitatory neurotransmitter, is also critically involved in multiple neurodevelopmental processes^3,55^. Our finding of higher Glx/tCr in the hippocampus in the ASD group is in line with changes in the amount of glutamate receptors described in the ASD literature^70^, with implications in the excitatory/inhibitory balance^11,64^ and important functions such as memory and learning^29,30,71^ which are impaired in ASD^32^.

Concerning tCho/tCr, we did not detect any significant alterations, unlike other studies^50,56,58^. However our results are consistent with previous studies that did not find alterations in (total) Cho in ASD participants regarding the insula^50^ (although this study did find differences in other regions), hippocampus-amygdala complex^57^, thalamus^63^ or putamen^63^.

It is important to uncover links between brain metabolites and ASD symptomatology and cognition^51,54,61,64^. Therefore, we investigated in an exploratory manner correlations between altered metabolite ratios and clinical and diagnostic measures (IQ, ADOS and ADI-R). In the insula, a weak negative correlation was found between tNAA/tCr and IQ measures (verbal subscale in ASD and performance IQ in the control group). In the hippocampus there was a moderate negative correlation between Glx ratios in the ASD group and total score of ADOS. The observed correlation of tNAA/tCho measures between the putamen and insula suggests that pathology in both structures may be associated. Given that it is known that these structures are strongly associated from the anatomical and functional points of view, this motivates future studies to investigate this relationship.

These data illustrate the complexity of comparing studies and analyzing clinical results from ^1^H-MRS in general. Although some consistency may be found for some metabolites such as tNAA, on the other hand there is some variability in results, that may be partly accounted for by differences in methodology or sample characteristics. For instance, differences in magnetic field strengths, sample size, age range, diagnostic criteria, regions, metabolite measurement, assessment of hemispheres individually or combined, sedation, medication, or demographic characteristics^72^, may substantially impact the results.

We adopted a multivoxel approach to address different structures in each individual at the same time point, with the exact same parameters. By using a small voxel size, we were able to study anatomically specific locations that were matched among individuals, enabling a more precise inter-individual analysis. Most ^1^H-MRS studies lack an effective spatial isolation of the insula, hippocampus or putamen, likely because of the larger voxel volumes studied using single-voxel approaches, that frequently included these regions in auditory or temporal cortex^73^, in a complex with the amygdala^52,57^, and in the basal ganglia^74^, respectively. Moreover, this was achieved at 3 T MRI scanner while the large majority of previous literature used lower static field strength systems (1.5 T)^3,9^. This was relevant to isolate small structures such as the hippocampus, which has been mainly studied as the combined hippocampal-amygdala complex, and to target rarely investigated ASD-affected regions such as the insula. The possibility of studying these areas is pertinent given the regional-dependency of metabolic alterations^64,75^. Another strength from our study is that we acquired all MRI images without sedating participants, unlike other studies^59,63,76,77^. Additionally, even though the inclusion of medicated participants could be a possible source of bias, here we observed the same findings when considering only non-medicated participants (see Supplementary Table S1).

In this study, we assessed Glx/tCr as an indicator of glutamatergic metabolism, due to the higher contribution of glutamate to the Glx ^1^H-MRS peak, as in other previous studies^3^. However, quantifying Glx using the current ^1^H-MRS protocol (i.e. TE = 135 ms) is challenging. Nevertheless, we used a basis set fitting approach which uses an a priori model of the expected peak shape and position as opposed to a simple peak based fitting (based on arbitrary model functions), mitigating quantification challenges. Also, a strict quality control was applied and excluded some Glx peaks from the statistical analysis (resulting in a lower sample size for the hippocampus and the thalamus, Supplementary Table S4). Yet, Glx/tCr results must be interpreted cautiously.

Typically, tCr is used as an internal reference value for calculating metabolite ratios^3,6,9^. However, there is regional and individual variability in creatine concentrations^4^, casting doubt on tCr as a suitable reference for metabolite measurements^66^. We additionally presented ratios to total choline as done previously^74,78,79^, since tCho/tCr was not different from TD in any of the regions in this study. While all main results were replicated using this reference, our findings should be interpreted in the light of the limitation of using ratios instead of absolute metabolite concentrations, as we cannot exclude that our references are altered.

In our study, we report tNAA (NAA + NAAG), which implies not only the contribution of NAA, which synthesis occurs in neuronal mitochondria^4^, but also of NAAG, which is synthesized from NAA and glutamate^64^. Since the signal accounts for both contributions, and NAA and NAAG play a distinct role in neural density and neurotransmission, respectively, lower tNAA signal in the ASD group could be due to decreased NAA, decreased NAAG, or both^64^.

## Conclusions

Here we provide evidence for lower tNAA/tCr in the insula and putamen and higher Glx/tCr in the hippocampus of children with ASD. These molecular changes point out these ratios as potential neurochemical biomarkers for altered metabolism and/or excitatory neurotransmission in ASD in a region-dependent manner. Our results might help understanding the differences that exist in specific brain structures, with potential future implications for the clinical monitoring and treatment of ASD patients.

## Methods

This work was conducted in accordance with the Declaration of Helsinki and got the approval from the Ethics Committee of the Faculty of Medicine of the University of Coimbra. All participants gave oral informed consent as well as their parents or legal representative gave written informed consent, after explanation of all the objectives of the study and procedures.

### Participants

We included 22 male participants with ASD, recruited from the Neurodevelopmental and Autism Unit from Child Developmental Centre, Paediatric Hospital, Centro Hospitalar e Universitário de Coimbra, Portugal. All participants were aged between 10 and 18 years old and met the diagnostic criteria determined in the *Diagnostic and Statistical Manual of Mental Disorders* (5th ed.; DSM-5^1^), and scored positive in the Autism Diagnostic Interview-Revised (ADI-R)^80^ and in the Autism Diagnostic Observation Schedule (ADOS)^81^. Participants were assessed at least twice a year by an experienced multidisciplinary team, including paediatricians specialized in neurodevelopment and psychologists. Five subjects who were taking medication (risperidone, n = 4; methylphenidate, n = 1) to control ASD symptoms continued treatment-as-usual. Additionally, 22 age- and gender-matched typically developing controls (CTRL), free from medication, recruited in district schools, entered in this study. The exclusion criteria for this study included the presence of diagnosed neurodevelopmental, psychiatric or neurological disorders other than ASD, genetic syndrome, serious learning disabilities (Full-Scale IQ inferior to 70 using the Portuguese abbreviated version of the Wechsler Intelligence Scale for Children, 3rd edition – WISC-III^82^ or by the Wechsler Adult Intelligence Scale, 3rd edition – WAIS-III^83^, when appropriate), and contraindications to magnetic resonance imaging. In addition, for the control participants, autism was ruled out by the Social Communication Questionnaire (SCQ)^84^ and by the Social responsiveness scale (SRS)^85^, both filled in by their parents.

Demographic and clinical data are presented in Table 1.

**Table 1:** Demographic and clinical data.

	ASD (*n* = 22)	CTRL (*n* = 22)
Chronological age (years, mean ± SD [range])	13.76 ± 2.08 [10.53–18.30]	13.56 ± 2.08 [9.55–18.21]
Sex (male: female)	22:0	22:0
Handedness (right: left)	22:0	19:2^a^
Full-scale IQ (score, mean ± SD [range])	91.23 ± 12.29 [70–114]	120.09 ± 19.65 [78–154]
Verbal IQ (score, mean ± SD [range])	89.68 ± 13.33 [66–117]	123.41 ± 20.60 [80–155]
Performance IQ (score, mean ± SD [range])	97.27 ± 16.85 [64–124]	111.18 ± 17.49 [84–149]
ADOS (score, mean ± SD [range])	13.45 ± 3.58 [8–19]	–
ADI-R (score, mean ± SD [range])	35.50 ± 10.00 [22–68]	–

*IQ* intelligence quotient, *ADOS* autism diagnostic observation schedule, *ADI*-*R* autism diagnostic interview-revised.

^a^Data available from 21 participants.

### Multivoxel proton magnetic resonance spectroscopy (^1^H-MRS)

MRI scanning was performed without sedation, at CIBIT/ICNAS (University of Coimbra) facilities, in a Siemens MAGNETOM TimTrio 3 T (Erlangen, Germany) scanner, provided with a 12-channel birdcage head coil. We started with the acquisition of anatomical data through a high-resolution 3D Magnetization Prepared Rapid Acquisition Gradient Echo (MPRAGE), with 160 slices, TR (repetition time) = 2300 ms, TE (echo time) = 2.98 ms, TI (inversion time) = 900 ms, FA (flip angle) = 9°, FOV (field-of-view) = 256 × 256 mm^2^, voxel size = 1 × 1 × 1 mm^3^.

Two-dimensional multivoxel ^1^H-MRS was applied in two different orientations to estimate metabolites concentration using a PRESS (Point RESolved Spectroscopy) sequence [TR = 1700 ms, TE = 135 ms, FA = 90°, 3 averages, 1024 points, FOV = 160 × 160 mm, VOI (volume-of-interest) = 80 × 80 × 15 mm, thickness = 15 mm]. A 12 × 12 matrix yielded a 13.3 × 13.3 × 15 mm nominal voxel size. This matrix was interpolated to 16 × 16 leading to a voxel nominal spatial resolution of 10.0 × 10.0 × 15 mm; 1.5 mL. Automatic shimming was applied to offset field inhomogeneities and achieve well-resolved peaks for the metabolites in study^86^ and chemical shift selective (CHESS) weak water suppression was executed at 50 Hz bandwidth. For the ^1^H-MRS slice prescription, the centre of the VOI was aligned with the brain midline. Specifically, for the insula, putamen, and thalamus, the slice was aligned to AC-PC in the sagittal view, and the bottom of the slice was aligned with the bottom of the corpus callosum. Next, in the coronal view, the structures of interest were centred on the ^1^H-MRS grid, aiming for symmetric and maximum voxel coverage for each structure in each hemisphere. For the hippocampus region, the ^1^H-MRS slab was aligned in the sagittal view along the longest axis of the hippocampus and then centred in the VOI for both hemispheres, ensuring symmetric and maximum coverage in each hemisphere. Outer-volume lipid suppression was achieved by applying saturation bands. The 2D-CSI acquisition followed a weighted phase encoding scheme with a Hamming filter. Each 2D ^1^H-MRS acquisition lasted around 3.7 min.

Participants were watching cartoons to keep them more relaxed and reduce motion during the experiment. Moreover, we used an eyetracking system [SensoMotoric Instruments (SMI), Teltow, Germany] to control positioning and movement throughout scanning.

**Figure 4:**
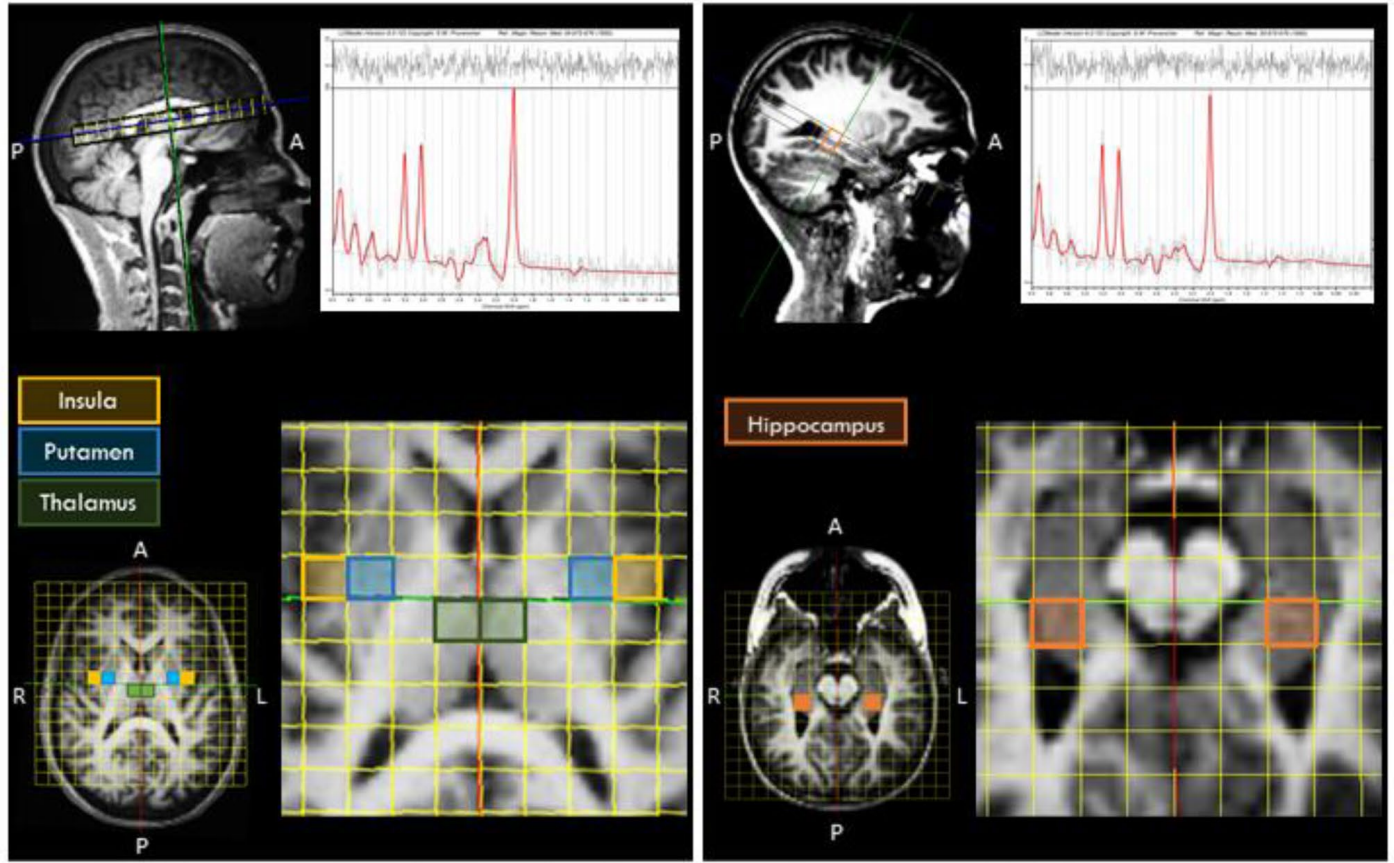
Example of the voxel selection, for each region, with an example of LCModel output for the insula (left spectrum) and for the hippocampus (right spectrum). *P* posterior, *A* anterior, *R* right, *L* left.

The ^1^H-MRS data were analysed with LCModel v. 6.3-1D (Stephen Provencher Inc., Oakville, Canada^87^), wherein we quantified metabolite levels in institutional units, without water-scaling or correcting for T1 and T2 water or metabolite relaxation times. LCModel uses an a priori model (basis set) of the expected peak shape and position as opposed to a simple peak based fitting (based on arbitrary model functions). The basis set used for fitting included alanine (Ala), creatine (Cr), phosphocreatine (PCr), glutamine (Gln), glutamate (Glu), glycerophosphocholine (GPC), phosphocholine (PCh), gluthatione (GSH), myo-inositol (mI), lactate (Lac), N-acetyl-aspartate (NAA), N-acetyl-aspartyl-glutamate (NAAG), scyllo-Inositol (Scyllo), and taurine (Tau). Some metabolite peaks were considered as pools of compounds: total choline (tCho), total n-acetyl-aspartate (tNAA) and Glx, as the sum of GPC + PCh, NAA + NAAG, and Glu + Gln, respectively. Ratios to total choline and total creatine (tCr, the pool of Cr + PCr) were computed. Please note that the analysis of ratios to total choline is secondary and serves as a test for replication, because Cho concentration frequently varies in psychiatric diseases. For each region, the mean of both hemispheres was considered. Complementary analyses between hemispheres are reported in Supplementary Table S4. Two researchers (A.D. and A.E.) selected one voxel per brain structure for each participant and hemisphere, namely insula, hippocampus, putamen, and thalamus. Voxels were selected considering the coverage of the region-of-interest while being standardized as much as possible among participants, aiming at the same exact anatomical region. The coverage of the brain regions was evaluated by visual inspection with the BrainCSI software (Jeffrey Yager, University of Iowa Carver College of Medicine, Iowa City, IA USA, https://www.nitrc.org/projects/braincsi). In Fig. 4 we illustrate an example of voxel selection, for each region in study, as well as an example of LCModel output spectrum.

Quantification of the proportion of grey matter (GM), white matter (WM) and cerebrospinal fluid (CSF) within each voxel was also carried out using an in-house developed CSI segmentation script resorting to SPM8 (Wellcome Trust Centre for Neuroimaging, Institute of Neurology, UCL, London, UK, https://www.fil.ion.ucl.ac.uk/spm/software/spm8/), with the VBM8 (Structural Brain Mapping Group, Department of Psychiatry, University of Jena, Germany) toolbox, run in MATLAB (R2019b, MathWorks, USA). Spectra were visually inspected and datasets with poor fitting or metabolite levels with Cramér-Rao lower bounds > 20% were excluded from the analyses. Other quality metrics, namely FWHM and SNR are provided in Supplementary Table S3.

### Statistics

Statistical tests were run in SPSS Statistics (IBM SPSS Statistics, IBM Corporation, Chicago, IL), version 27. We first characterized both groups, using descriptive statistics. Then, we evaluated data normality with the Shapiro–Wilk test. Participants’ characteristics and metabolite concentrations in each brain region were compared between groups by computing independent samples *t* test or its non-parametric equivalent Mann–Whitney *U*, where appropriate. Handedness was compared using Fisher’s exact test. We report exact p-values for non-parametric comparisons. Additionally, we applied Pearson’s correlation coefficient or Spearman’s rho to investigate correlations between intellectual quotient measures and metabolite levels in each group. For the ASD group alone, we also investigated possible correlations between the diagnostic scores (ADOS/ADI-R) and the levels of altered metabolites in the regions of interest. None of these correlations were corrected for multiple comparisons and are, thus, exploratory. We adopted a significance level of 0.05.

### Supplementary Information

Supplementary Tables.

## Data Availability

The datasets generated during and/or analysed during the current study are available from the corresponding author on reasonable request.
