# Supplementary material for: Neurochemical differences in core regions of the autistic brain: a multivoxel 1H-MRS study in children
Source: Sci Rep. 2024 Jan 29;14:2374. doi: 10.1038/s41598-024-52279-x (PMC10824733; doi:10.1038/s41598-024-52279-x)
Supplement: Supplementary file 1 — Supplementary Tables. [file 41598_2024_52279_MOESM1_ESM.docx]

Supplementary Table S1: Statistical analysis comparing metabolite ratios to creatine, between non-medicated ASD and CRTL, in each region of interest.

|  | tNAA/tCr | tCho/tCr | Glx/tCr |
| --- | --- | --- | --- |
| Insula | **t(37) = -2.345, p = 0.025** (n_ASD_ = 17, n_CTRL_ = 22) | U = 182.0, p = 0.887 (n_ASD_ = 17, n_CTRL_ = 22) | t(37) = 0.069, p = 0.946 (n_ASD_ = 17, n_CTRL_ = 22) |
| Hippocampus | t(21.9) = -0.469, p = 0.644 (n_ASD_ = 17, n_CTRL_ = 22) | t(24.9) = -0.510, p = 0.614 (n_ASD_ = 17, n_CTRL_ = 22) | **t(32) = 2.588, p = 0.014**  (n_ASD_ = 14, n_CTRL_ = 20) |
| Putamen | **U = 112.5, p = 0.053** (n_ASD_ = 17, n_CTRL_ = 21) | t(36) = 0.281, p = 0.780 (n_ASD_ = 17, n_CTRL_ = 21) | t(36) = 0.191, p = 0.850 (n_ASD_ = 17, n_CTRL_ = 21) |
| Thalamus | t(27.4) = 1.711, p = 0.098  (n_ASD_ = 17, n_CTRL_ = 22) | U = 184.0, p = 0.932 (n_ASD_ = 17, n_CTRL_ = 22) | t(15) = 0.403, p = 0.693 (n_ASD_ = 6, n_CTRL_ = 11) |

Supplementary Table S2: Tissue proportion in the studied voxels

|  | ASD (*n* = 22) | CTRL (*n* = 22) | Statistics |
| --- | --- | --- | --- |
| Insula | | |  |
| WM (%, mean ± SD [range]) | 16.00 ± 7.57 [3.94-32.86] | 16.17 ± 9.86 [3.50-44.27] | U = 220.0, p = 0.617 |
| GM (%, mean ± SD [range]) | 80.06 ± 6.72 [64.80-91.86] | 80.14 ± 8.84 [54.77-91.60] | U = 223.5, p = 0.671 |
| CSF (%, mean ± SD [range]) | 3.94 ± 2.09 [0.47-8.96] | 3.69 ± 1.98 [0.97-7.83] | t(42) = 0.404, p = 0.688 |
| Hippocampus | | |  |
| WM (%, mean ± SD [range]) | 43.97 ± 7.55 [31.11-58.39] | 44.77 ± 9.45 [23.15-60.83] | t(42) = -0.308, p = 0.760 |
| GM (%, mean ± SD [range]) | 53.85 ± 6.78 [40.10-64.55] | 52.30 ± 8.80 [37.82-72.99] | t(42) = 0.656, p = 0.515 |
| CSF (%, mean ± SD [range]) | 2.18 ± 1.80 [0.17-7.63] | 2.94 ± 1.67 [0.40-6.81] | **U = 158.0, p =0.049** |
| Putamen | | |  |
| WM (%, mean ± SD [range]) | 27.10 ± 8.39 [11.69-44.36] | 32.46 ± 12.35 [10.54-64.40]^a^ | t(41) = -1.671, p = 0.102 |
| GM (%, mean ± SD [range]) | 72.90 ± 8.39 [55.65-88.31] | 67.53 ± 12.38 [35.40-89.47]^a^ | t(41) = 1.671, p = 0.102 |
| CSF (%, mean ± SD [range]) | 0.00 ± 0.01 [0.00-0.04] | 0.01 ± 0.04 [0.00-0.20]^a^ | U = 230.0, p = 0.744 |
| Thalamus | | |  |
| WM (%, mean ± SD [range]) | 17.69 ± 7.75 [7.55-34.87] | 13.08 ± 6.76 [3.77-26.13] | **t(42) = 2.103, p = 0.042** |
| GM (%, mean ± SD [range]) | 74.21 ± 6.62 [56.90-83.04] | 76.15 ± 8.07 [60.37-88.23] | t(42) = -0.871, p = 0.389 |
| CSF (%, mean ± SD [range]) | 8.10 ± 5.40 [2.75-23.98] | 10.77 ± 6.91 [1.67-25.34] | U = 191.0, p = 0.239 |

Abbreviations: WM, White Matter; GM, Gray Matter; CSF, Cerebrospinal Fluid

^a^ Data available from 21 participants

Supplementary Table S3: MRS quality parameters in the studied voxels

|  | ASD (*n* = 22) | CTRL (*n* = 22) | Statistics |
| --- | --- | --- | --- |
| Insula | | |  |
| FWHM (ppm, mean ± SD [range]) | 0.061 ± 0.012 [0.041-0.086] | 0.055 ± 0.013 [0.033-0.085] | t(42) = 1.668, p = 0.103 |
| SNR (mean ± SD [range]) | 8.82 ± 1.52 [6.50-11.50] | 10.52 ± 2.82 [7.50-19.00] | **U = 143.5, p = 0.019** |
| Hippocampus | | |  |
| FWHM (ppm, mean ± SD [range]) | 0.055 ± 0.012 [0.038-0.081] | 0.052 ± 0.014 [0.031-0.081] | t(42) = 0.607, p = 0.547 |
| SNR (mean ± SD [range]) | 6.73 ± 1.30 [4.50-9.50] | 7.52 ± 1.72 [5.00-10.50] | t(42) = -1.731, p = 0.091 |
| Putamen | | |  |
| FWHM (ppm, mean ± SD [range]) | 0.063 ± 0.012 [0.043-0.091] | 0.056 ± 0.013 [0.040-0.086] ^a^ | t(41) = 1.709, p = 0.095 |
| SNR (mean ± SD [range]) | 8.16 ± 1.76 [5.50-12.00] | 9.90 ± 2.12 [6.50-14.00] ^a^ | **t(41) = -2.938, p = 0.005** |
| Thalamus | | |  |
| FWHM (ppm, mean ± SD [range]) | 0.060 ± 0.012 [0.040-0.080] | 0.058 ± 0.011 [0.040-0.080] | t(42) = 0.711, p = 0.481 |
| SNR (mean ± SD [range]) | 6.45 ± 1.50 [4.00-10.50] | 6.55 ± 1.42 [4.00-8.50] | t(42) = -0.207, p = 0.837 |

Abbreviations: FWHM, Full Width at Half Maximum; SNR, Signal-to-Noise Ratio

^a^ Data available from 21 participants

Supplementary Table S4: Statistical analysis comparing metabolite ratios to creatine separately for the right and left hemisphere, between ASD and CRTL, in each region of interest.

|  | Right hemisphere Statistics | Left hemisphere Statistics |
| --- | --- | --- |
| Insula | | |
| tNAA/tCr | **t(42) = -2.410, p = 0.020** (n_ASD_ = 22, n_CTRL_ = 22) | **t(42) = -2.062, p = 0.045** (n_ASD_ = 22, n_CTRL_ = 22) |
| tCho/tCr | t(42) = 0.915, p = 0.365 (n_ASD_ = 22, n_CTRL_ = 22) | U = 231.0, p = 0.796 (n_ASD_ = 22, n_CTRL_ = 22) |
| Glx/tCr | t(42) = 0.837, p = 0.407 (n_ASD_ = 22, n_CTRL_ = 22) | U = 204.5, p = 0.379 (n_ASD_ = 22, n_CTRL_ = 22) |
| Hippocampus | | |
| tNAA/tCr | t(36.4) = -0.539, p = 0.593 (n_ASD_ = 22, n_CTRL_ = 22) | t(42) = -1.019, p = 0.314 (n_ASD_ = 22, n_CTRL_ = 22) |
| tCho/tCr | t(33.8) = 0.162, p = 0.872 (n_ASD_ = 22, n_CTRL_ = 22) | t(42) = -1.727, p = 0.091 (n_ASD_ = 22, n_CTRL_ = 22) |
| Glx/tCr | t(27) = 1.842, p = 0.077 (n_ASD_ = 12, n_CTRL_ = 17) | t(26) = 0.759, p = 0.455 (n_ASD_ = 14, n_CTRL_ = 14) |
| Putamen | | |
| tNAA/tCr | **t(41) = -2.472, p = 0.018** (n_ASD_ = 22, n_CTRL_ = 21) | t(41) = -1.388, p = 0.172 (n_ASD_ = 22, n_CTRL_ = 21) |
| tCho/tCr | t(41) = 1.139, p = 0.261 (n_ASD_ = 22, n_CTRL_ = 21) | t(41) = -0.501, p = 0.619 (n_ASD_ = 22, n_CTRL_ = 21) |
| Glx/tCr | t(41) = 0.252, p = 0.802 (n_ASD_ = 22, n_CTRL_ = 21) | t(39) = -0.969, p = 0.338 (n_ASD_ = 20, n_CTRL_ = 21) |
| Thalamus | | |
| tNAA/tCr | t(32.7) = 1.565, p = 0.127 (n_ASD_ = 22, n_CTRL_ = 22) | t(41) = 0.500, p = 0.619 (n_ASD_ = 22, n_CTRL_ = 21) |
| tCho/tCr | t(42) = -0.185, p = 0.854 (n_ASD_ = 22, n_CTRL_ = 22) | t(41) = 0.689, p = 0.494 (n_ASD_ = 22, n_CTRL_ = 21) |
| Glx/tCr | t(12) = 0.643, p = 0.532 (n_ASD_ = 5, n_CTRL_ = 9) | t(12) = 0.021, p = 0.984  (n_ASD_ = 6, n_CTRL_ = 8) |
